# Predictive factors for the development of depression in children and adolescents: a clinical study

**DOI:** 10.3389/fpsyt.2024.1460801

**Published:** 2024-10-14

**Authors:** Hong Zhang, Peilin Yu, Xiaoming Liu, Ke Wang

**Affiliations:** ^1^ The Second Clinical Medical School, Xuzhou Medical University, Xuzhou, Jiangsu, China; ^2^ Department of Biostatistics, School of Public Health, Xuzhou Medical University, Xuzhou, Jiangsu, China; ^3^ Xuzhou Children’s Hospital Affiliated to Xuzhou Medical University, Xuzhou, Jiangsu, China; ^4^ Center for Medical Statistics and Data Analysis, Xuzhou Medical University, Xuzhou, Jiangsu, China; ^5^ Jiangsu Engineering Research Center of Biological Data Mining and Healthcare Transformation, Xuzhou Medical University, Xuzhou, Jiangsu, China; ^6^ Research Center for Psychological Crisis Prevention and Intervention of college in Jiangsu Province, Xuzhou, Jiangsu, China

**Keywords:** logistic regression, NCHS, adolescents, depression, nomogram, prediction

## Abstract

**Background:**

The prevalence of depression among adolescents has been gradually increasing with the COVID-19 pandemic, and the purpose of this study was to develop and validate logistic regression models to predict the likelihood of depression among 6-17 year olds.

**Methods:**

We screened participants from the National Center for Health Statistics (NCHS) in 2022. Independent risk factors were identified via univariate logistic regression analyses and least absolute shrinkage and selection operator (LASSO) for feature screening. Area under the curve (AUC) and decision curve analysis (DCA) were used to compare the predictive performance and clinical utility of these models. In addition, calibration curves were used to assess calibration.

**Results:**

Multivariate logistic regression analyses revealed that risk factors for depression included girls, higher age, treated/judged based on race/ethnicity, ever lived with anyone mentally ill, experienced as a victim of/witnessed violence, and ever had autism, ever had attention-deficit disorder (ADD), etc. Afterwards, the results are visualized using a nomogram. The AUC of the training set is 0.731 and the AUC of the test set is 0.740. Also, the DCA and calibration curves demonstrate excellent performance.

**Conclusion:**

Validated nomogram can accurately predict the risk of depression in children and adolescents, providing clues for clinical practitioners to develop targeted interventions and support.

## 1 Introduction

Depression is among the more common psychiatric disorders in children and adolescents. At any given time, nearly 3% of youth worldwide are reported to have a depression (1). Before the COVID-19 pandemic, the prevalence of major depression among adolescents was reported to be about 13%–15% (2, 3). A recent meta-analysis found that around 1 in 4 youth had clinically significant depressive symptoms during the COVID-19 pandemic, with higher rates associated with older age and female sex; it also found the prevalence of symptoms to be higher later during the pandemic period (4). Depression is a leading cause of disability and a major contributor to the overall global burden of disease (5). Nearly 30% of youth with major depression reported some form of suicidality in the past year, and more than 10% reported a suicide attempt. Additionally, depression in young people also has widespread negative impacts on psychosocial functioning including lower educational attainment, higher welfare dependence and unemployment in adulthood (6–8). There is also evidence to suggest impacts on future interpersonal difficulties, including marital functioning (9), increased loneliness (10) and a greater need for social support (11).

Although more than 40% of people with depression experience onset before adulthood, depression remains undetected in many adolescents worldwide, and most are untreated (12–14). Only 34% of adolescents with major depression were reported to receive disorder-specific treatment, and only 35% received treatment from the mental health sector (15). Many clinicians consistently report a lack of confidence in their ability to care for adolescents with depression (16). Furthermore, preventing the onset or recurrence of depression in childhood and adolescence has been reported to promote improved functioning in adulthood (17). Therefore, it is critical to identify individuals who were likely to develop depression as early as possible and to prevent its onset. However, predicting which individuals will experience depression and anxiety in adolescence remains an extremely difficult task. There is increasing recognition of the immense complexity of psychopathology, necessitating shifting away from simple etiological models and toward a complex dynamic systems perspective that recognizes that mental disorders arise from the interplay of numerous interacting components on multiple levels of analysis (18).

In a research review on child and adolescent psychiatry, the use of ML in the prediction of depression is demonstrated (19). Early depression questionnaire data were identified with the help of cross-validated neural network studies (19, 20). In addition, more recent studies have begun to focus on the collection of multimodal data, such as facial expressions, speech features, and magnetic resonance imaging (MRI) examination information, to improve early disease diagnosis and symptom prediction (21–23). Despite these significant advances, there are still some limitations in terms of different populations and clinical features and further research is urgently needed to enhance the generalisability and adaptability of the models (24). This study focuses on investigating the factors associated with the occurrence of depression among children and adolescents aged 6-17 years in a clinical prediction study. To predict the likelihood of developing depression, logistic regression and nomogram techniques will be utilized. Logistic regression allowed us to examine the relationship between multiple independent variables and the binary outcome variable. Least Absolute Shrinkage and Selection Operator (LASSO) regression imposes an L1 penalty by adjusting the value of λ, which results in an additional contraction of the absolute value of the logistic regression coefficients. This approach not only effectively retains the most predictive variables, but also reduces overfitting of the model, which improves the generalization ability of the model and makes the final model more accurate (25–27).

By analyzing the data collected from the study participants, we will be able to determine the strength and direction of the association between each predictor and depression. Besides, a nomogram will be constructed to provide a visual representation of the prediction model that can estimate an individual’s risk of developing depression based on specific characteristics of the individual and identified predictors. In addition, the findings from this study will provide valuable insights for healthcare professionals, educators, and policymakers to develop targeted interventions and support systems for at-risk individuals.

## 2 Materials and methods

### 2.1 Data and participants

**Figure 1:**
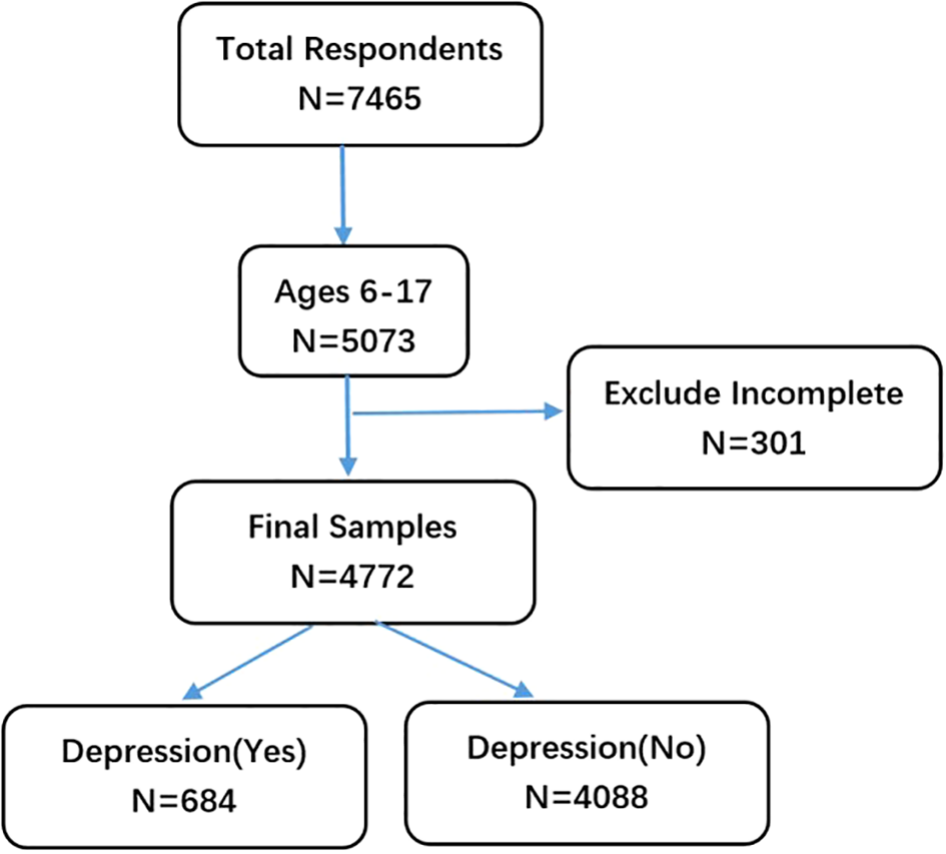
Flow chart for filtering participants in NHIS.

We obtained data from the National Health Interview Survey (NHIS) conducted in 2022. NHIS is an annual survey administered by the National Center for Health Statistics (NCHS), aiming to collect health-related information on the civilian noninstitutionalized population of the United States. It has been widely utilized to estimate disease prevalence nationwide. The research ethics review board of NCHS approved NHIS, ensuring its compliance with ethical standards (28, 29). In order to minimize respondent burden and improve data quality, NHIS underwent a redesign in 2022. During sampling, one adult aged ≥18 years old and one child aged ≤17 years old (if applicable) were randomly selected from each household. Information regarding children was gathered from parents or responsible adults knowledgeable about their healthcare needs. For this study, only children’s data were retrieved for analysis purposes. In 2022, a total of 7,464 questionnaires were collected by NHIS, including 5,073 questionnaires from children and adolescents aged 6 to 17. 301 questionnaires with high deletion rate were deleted, and a total of 4772 subjects were included in this study. The detailed process of participant selection is shown in **Figure 1**.

### 2.2 Measurements and operational definitions

#### 2.2.1 Depression

The Child Functioning Module (CFM) is a 24-item scale developed by the Washington Group on Disability Statistics in collaboration with the United Nations Children’s Fund (UNICEF) to measure the frequency of depression in children (30–32). The CFM was cognitively tested internationally across six countries and is designed to capture a child’s functional status, reflecting advances in the conceptualization of disability using the World Health Organization’s (WHO) International Classification of Functioning, Disability, and Health (33). Parents are asked to report on the frequency with which their child seems “very sad or depressed” with response options of “daily,” “weekly,” “monthly,” “a few times a year,” or “never”. For this study, children were categorized as having current symptoms of depression based on a frequency response of “daily” or “weekly” or “Monthly” to either question. These questions are included in a module for children 5–17 years of age which focuses on the domains of seeing, hearing, mobility, dexterity, self-care, communication, learning, cognition, affect, playing, behavior, and peer relationships (34).

#### 2.2.2 Data collection

Demographic characteristics were examined for both children and families. These factors included the gender of the child (boy and girl), age (6-17 years old), race/ethnicity (non-Hispanic White, non-Hispanic Black, non-Hispanic other, Hispanic), level of urbanization (35) (large central or fringe metropolitan area, medium or small metropolitan area, nonmetropolitan area), family income as a percentage of the federal poverty level (<100%, 100%–199%, 200–399%, ≥400%), and highest educational attainment of any parent residing in the household (high school education or less, associate degree or some college education, bachelor’s degree or higher). The number of adults in the household (1 adult, ≥2 adults), the number of children in the household (1 child, ≥2 children). The definition of physical activity is to exercise, participate in sports, or engage in at least 60 minutes of physical activity daily or most days (yes or no). Stressful life events of the child or his/her family members were included in the dataset of 2022. Relevant information included treated/judged based on race/ethnicity (yes and no), lifetime of lacking basic needs (yes and no), experience as a victim of/witnessed violence (yes and no), bullied by others (yes and no), ever lived with a parent who was incarcerated (yes and no), ever lived with anyone mentally ill (yes and no), and ever lived with anyone with an alcohol problem (yes and no). The study also included Developmental and Learning Disabilities, such as Ever had learning disabilities (yes and no), Ever had developmental delay (yes and no), Ever had autism (yes and no), Ever had intellectual disability (yes and no), Ever had Attention-Deficit/Hyperactivity Disorder (ADHD) or Attention-Deficit Disorder (ADD) (yes and no). Indicators are also included in terms of physical health status, ever had asthma (yes and no), the Washington Group Short Set Composite Disability Indicator (yes and no), In the General health status, individuals who report their health as ‘Excellent’, ‘Very Good’, or ‘Good’ are categorized as having a ‘good’ health status, while those reporting any other response are classified as having a ‘poor’ health status.

### 2.3 Statistical analysis

The dataset collected from the 2022 NHIS was randomly divided into training and test cohorts at a ratio of 7:3, and the variables were compared. Non-normal data were presented as median (interquartile ranges). In the univariate analysis, chi-square test or Fisher’s exact test was used to analyze the categorical variables, while the rank-sum test was used to examine the continuous variables. In the training cohort, in order to overcome the limitations of traditional stepwise selection and to prevent overfitting, we chose to use LASSO for the simplification of the model, filtering out the variables with smaller absolute values of the coefficients in the model and obtaining the more strongly independent risk factors in it. The following is the formula for coefficient estimation:


$$\hat{\beta }=\text{argmin}\sum_{i=1}^{n}\left\{y_{i}\eta_{\beta }\left(X^{i}\right)-\text{ln}\left\{1+\text{exp}\left[\eta_{\beta }\left(X^{i}\right)\right]\right\}\right\}+\lambda \sum_{j=1}^{p}\left|\beta_{j}\right|$$

The parameter λ in LASSO regression indicates the complexity of the model, and the larger the value, the fewer the variables included in the model. Then, we applied multivariate logistic regression analysis to identify independent predictors of depression in adolescents. Finally, in order to combine multiple factors for individualized prediction, we create a predictive column-line diagram of depression to aid clinical decision-making. The performance of the nomogram was assessed using the receiver operating characteristic (ROC) curve and calibration curve, with the area under the ROC curve (AUC) ranging from 0.5 (no discriminant) to 1 (complete discriminant). A decision curve analysis (DCA) was also performed to determine the net benefit threshold of prediction. Results with a *P* -value of <0.05 were considered significant. All statistical analyses were performed using the R software (version 4.2.2).

## 3 Results

### 3.1 Patient characteristics

The baseline characteristics of the study population are shown in **Supplementary Table 1**. The comparison of variables in the training cohort (70%) and test cohort (30%) is shown in **Table 1**. The results of hypothesis tests were consistent between the training and test cohorts, except for the variables of family income to poverty ratio (training cohort: *P*=0.013, test cohort: *P*=0.864), Race/ethnicity (training cohort: *P*<0.001, test cohort: *P*=0.193), the number of adults(training cohort: *P*=0.304, test cohort: *P*=0.035), the number of children(training cohort: *P*=0.239, test cohort: *P*=0.040), head discomfort(training cohort: *P*=0.068, test cohort: *P*=0.007).

**Table 1:** Comparison of variables between groups of different outcomes in training and internal test cohorts.

Characteristics	Training Cohort			Internal Test Cohort		
	No, N = 3,321^1^	Yes, N = 515^1^	*P* ^2^	No, N = 1,394^1^	Yes, N = 250^1^	*P* ^2^
**Urbanization level**			0.117			0.579
Medium and small metro	439 (13%)	74 (14%)		190 (14%)	40 (16%)	
Large metropolitan	993 (30%)	173 (34%)		416 (30%)	75 (30%)	
Non-metropolitan	1,889 (57%)	268 (52%)		788 (57%)	135 (54%)	
**Family income to poverty ratio**			0.013			0.864
<100%	343 (10%)	75 (15%)		161 (12%)	33 (13%)	
100–199%	704 (21%)	89 (17%)		263 (19%)	49 (20%)	
200–399%	987 (30%)	149 (29%)		422 (30%)	74 (30%)	
≥400%	1,287 (39%)	202 (39%)		548 (39%)	94 (38%)	
**Sex**			<0.001			<0.001
Girls	1,591 (48%)	309 (60%)		614 (44%)	151 (60%)	
Boys	1,728 (52%)	206 (40%)		780 (56%)	99 (40%)	
**Age, years**			<0.001			<0.001
Median (IQR)	11.0 (8.0, 15.0)	13.0 (9.0, 16.0)		11.0 (8.0, 14.8)	14.0 (10.0, 15.0)	
**Race/ethnicity**			<0.001			0.193
Non-Hispanic Black	1,581 (48%)	321 (62%)		676 (48%)	139 (56%)	
Hispanic	370 (11%)	42 (8%)		143 (10%)	19 (7%)	
Non-Hispanic White	913 (27%)	91 (18%)		372 (27%)	60 (24%)	
Non-Hispanic other	457 (14%)	61 (12%)		203 (15%)	32 (13%)	
**The number of adults**			0.304			0.035
1 adult	472 (14%)	82 (16%)		167 (12%)	42 (17%)	
≥2 adults	2,849 (86%)	433 (84%)		1,227 (88%)	208 (83%)	
**The number of children**			0.239			0.040
1 child	1,315 (40%)	218 (42%)		545 (39%)	115 (46%)	
≥2 children	2,006 (60%)	297 (58%)		849 (61%)	135 (54%)	
**Highest level of education^3^**			0.075			0.308
Associate degree or some college	868 (26%)	159 (31%)		371 (27%)	78 (31%)	
High school or less	727 (22%)	102 (20%)		301 (22%)	52 (21%)	
Bachelor’s degree or higher	1,722 (52%)	254 (49%)		719 (52%)	119 (48%)	
**Behavior^4^**			0.685			0.515
Yes	411 (12%)	67 (13%)		185 (13%)	37 (15%)	
No	2,910 (88%)	448 (87%)		1,209 (87%)	213 (85%)	
**Physical activity**			<0.001			<0.001
Yes	1,999 (67%)	264 (55%)		856 (69%)	137 (57%)	
No	964 (33%)	218 (45%)		387 (31%)	104 (43%)	
**Head discomfort^5^**			0.068			0.007
Yes	201 (6%)	42 (8%)		77 (6%)	25 (10%)	
No	3,120 (94%)	473 (92%)		1,317 (94%)	225 (90%)	
**Unfairer**			<0.001			<0.001
Yes	156 (5%)	60 (12%)		59 (4%)	28 (12%)	
No	3,065 (95%)	448 (88%)		1,303 (96%)	215 (88%)	
**Lacking basic needs**			<0.001			<0.001
Yes	105 (3%)	39 (8%)		39 (3%)	23 (9%)	
No	3,141 (97%)	472 (92%)		1,330 (97%)	223 (91%)	
**Putdown**			<0.001			<0.001
Yes	127 (4%)	64 (13%)		50 (4%)	36 (15%)	
No	3,107 (96%)	441 (87%)		1,312 (96%)	208 (85%)	
**Living with the addict^6^**			<0.001			<0.001
Yes	269 (8%)	96 (19%)		122 (9%)	58 (24%)	
No	2,969 (92%)	414 (81%)		1,248 (91%)	186 (76%)	
**Living with the mental^7^**			<0.001			<0.001
Yes	237 (7%)	118 (23%)		105 (8%)	62 (25%)	
No	3,000 (93%)	390 (77%)		1,266 (92%)	184 (75%)	
**Separate with the jailers^8^**			<0.001			<0.001
Yes	186 (6%)	65 (13%)		95 (7%)	36 (15%)	
No	3,058 (94%)	447 (87%)		1,277 (93%)	210 (85%)	
**Victim of/witnessed violence**			<0.001			<0.001
Yes	176 (5%)	78 (15%)		69 (5%)	39 (16%)	
No	3,068 (95%)	429 (85%)		1,300 (95%)	206 (84%)	
**COVID-19**			<0.001			0.024
Yes	1,186 (36%)	226 (44%)		486 (35%)	106 (42%)	
No	2,114 (64%)	287 (56%)		904 (65%)	144 (58%)	
**Learning disability**			<0.001			<0.001
Yes	247 (7%)	80 (16%)		116 (8%)	39 (16%)	
No	3,070 (93%)	435 (84%)		1,273 (92%)	210 (84%)	
**Developmental delay**			<0.001			<0.001
Yes	132 (4%)	50 (10%)		66 (5%)	27 (11%)	
No	3,189 (96%)	465 (90%)		1,325 (95%)	221 (89%)	
**Autism**			<0.001			<0.001
Yes	96 (3%)	48 (9%)		44 (3%)	22 (9%)	
No	3,222 (97%)	465 (91%)		1,344 (97%)	227 (91%)	
**Intellectual disability**			<0.001			<0.001
Yes	44 (1%)	19 (4%)		24 (2%)	14 (6%)	
No	3,275 (99%)	496 (96%)		1,367 (98%)	235 (94%)	
**ADHD^9^**			<0.001			<0.001
Yes	342 (10%)	125 (24%)		136 (9.8%)	66 (27%)	
No	2,970 (90%)	390 (76%)		1,253 (90%)	183 (73%)	
**Asthma**			0.009			0.006
Yes	394 (12%)	82 (16%)		160 (11%)	44 (18%)	
No	2,926 (88%)	432 (84%)		1,234 (89%)	205 (82%)	
**Health Status**			<0.001			<0.001
Good	3,259 (98%)	473 (92%)		1,369 (98%)	230 (92%)	
Poor	62 (2%)	42 (8%)		25 (2%)	20 (8%)	

^1^n (%).

^2^Wilcoxon rank sum test; Pearson’s Chi-squared test.

^3^The highest level of education refers to the highest level of education among all sample child’s parents.

^4^The Washington Group Short Set Composite Disability Indicator.

^5^Ever headache, vomit, blurred vision, or mood change after blow to head.

^6^Ever lived with anyone with alcohol/drug problem.

^7^Ever lived with anyone mentally ill/severely depressed.

^8^Ever separated from parent who was incarcerated.

^9^Had Attention-Deficit/Hyperactivity Disorder (ADHD) or Attention-Deficit Disorder (ADD).

### 3.2 Predictive model

**Figure 2:**
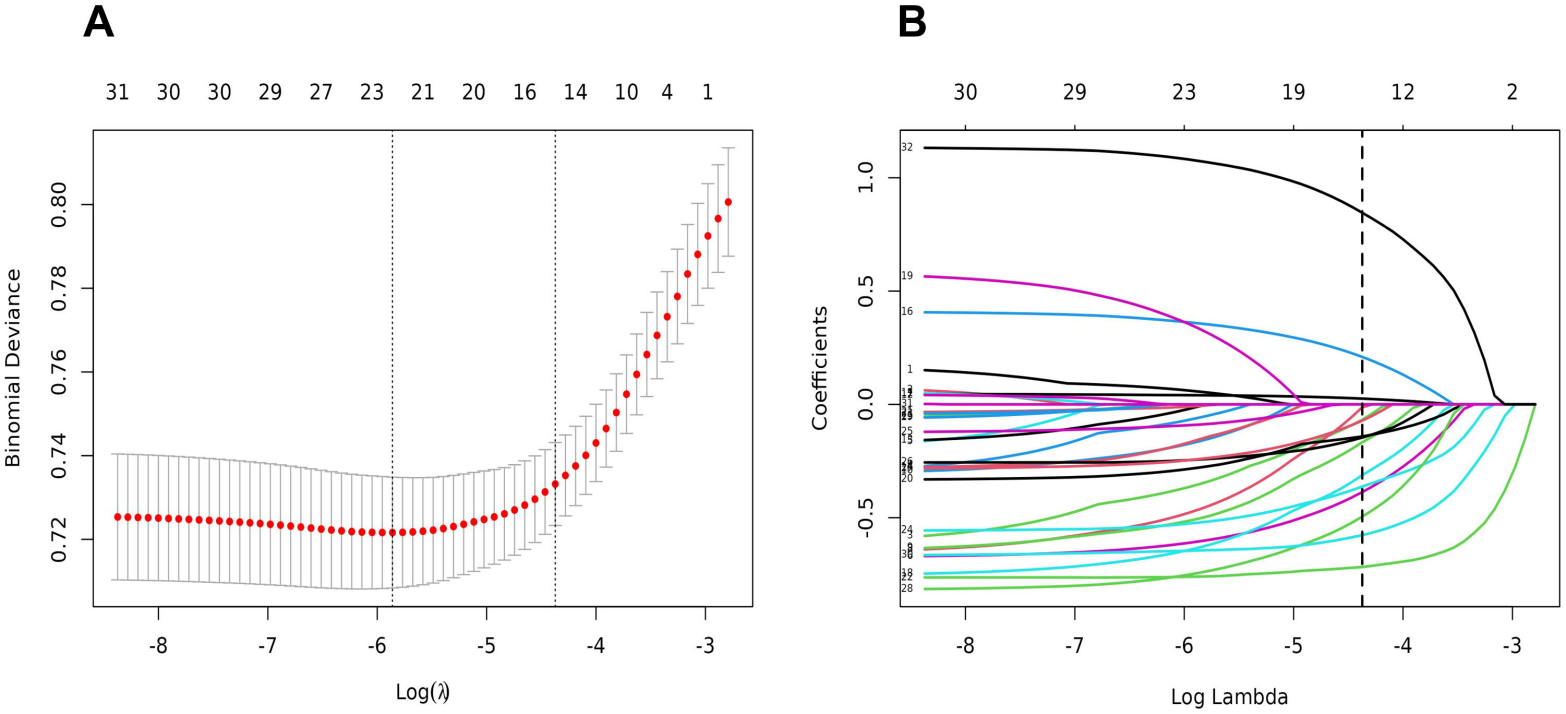
Feature selection using the LASSO binary logistic regression model. **(A)** Optimal parameter (lambda) selection in the LASSO model used fivefold cross-validation via minimum criteria. The partial likelihood deviance (binomial deviance) curve was plotted versus log(lambda). Dotted vertical lines were drawn at the optimal values by using the minimum criteria and the 1 SE of the minimum criteria (the 1-SE criteria). **(B)** LASSO coefficient profiles of the 26 features. A coefficient profile plot was produced against the log(lambda) sequence. Vertical line was drawn at the value selected using fivefold cross-validation, where optimal lambda resulted in five features with nonzero coefficients.

We incorporated 26 baseline characteristics, after which we screened for optimal factors with non-zero coefficients by building a LASSO regression (coefficients are displayed in **Supplementary Table 2**, and the distribution of coefficients is shown in **Figure 2**). Cross-validation error plots are shown for 1-SE and optimal λ. We chose 1-SE as it usually yields a more stable and simpler model, increases tolerance, avoids overfitting, and also contributes to model interpretability and computational efficiency. Finally, the highest variable importance included 14 potential predictors such as gender, age, mental life, autism and health status.

In LASSO regression, the coefficients of the features are selected after compression and it may not be clear whether these features have an important independent role in practical applications. However, univariate logistic regression assesses the relationship between each feature and the response variable independently of other features, and also provides quantification of the specific effects of these features, helping to understand their actual importance and increasing the accuracy and reliability of the model assessment. In addition, univariate logistic regression provides simple models of the effects of features that help understand the effect of each feature on the target variable and are easy to interpret. To verify the rationality of the selected variables, we performed a univariate logistic regression in the training set. The results are shown in **Table 2**.

**Table 2:** Results of univariate logistic regression.

Predictor	β_b_	SE	Waldχ^2^	P	OR	OR(95% CI)
**Intercept**	-2.691	0.359	-7.481	<0.001	0.06	(0.03, 0.13)
Urbanization level						
Large metropolitan	-0.006	0.101	-0.064	0.949	0.99	(0.81, 1.22)
Nonmetropolitan	-0.073	0.143	-0.512	0.609	0.92	(0.70, 1.23)
Family income to poverty ratio						
100–199%	-0.351	0.166	-2.115	0.034	0.70	(0.50, 0.97)
200–399%	-0.192	0.163	-1.175	0.240	0.82	(0.59, 1.13)
≥400% – <100%	-0.132	0.176	-0.752	0.452	0.87	(0.62, 1.23)
Sex						
Boys	-0.746	0.093	-7.99	<0.001	0.47	(0.39, 0.56)
**Age, years**	0.053	0.013	3.884	<0.001	1.05	(1.02, 1.08)
Race/ethnicity						
Hispanic	0.174	0.185	0.938	0.348	1.19	(0.82, 1.71)
Non-Hispanic White	0.669	0.176	3.784	<0.001	1.95	(1.38, 2.76)
Non-Hispanic other	0.375	0.202	1.855	0.064	1.45	(0.97, 2.16)
The number of adults						
≥2 adults	-0.011	0.130	-0.085	0.932	0.98	(0.76, 1.27)
The number of children						
≥2 children	-0.001	0.093	-0.013	0.989	0.99	(0.83, 1.20)
Highest level of education						
High school or less	-0.182	0.135	-1.35	0.177	0.83	(0.63, 1.08)
Bachelor’s degree or higher	-0.180	0.114	-1.578	0.115	0.83	(0.66, 1.04)
Behavior						
Yes – No	-0.130	0.129	-1.005	0.315	0.87	(0.68, 1.13)
Physical activity						
No – Yes	0.387	0.092	4.193	<0.001	1.47	(1.22, 1.76)
Head discomfort						
No – Yes	-0.144	0.164	-0.881	0.378	0.86	(0.62, 1.19)
Unfairer						
Yes – No	0.754	0.162	4.645	<0.001	2.12	(1.54, 2.92)
Lacking basic needs						
Yes – No	-0.259	0.229	-1.131	0.258	0.77	(0.49, 1.20)
Putdown						
Yes – No	0.379	0.185	2.045	0.041	1.46	(1.01, 2.10)
Living with the addict						
Yes – No	0.163	0.158	1.037	0.300	1.17	(0.86, 1.60)
Living with the mental						
Yes – No	0.694	0.140	4.951	<0.001	2.00	(1.52, 2.63)
Separate with the jailers						
Yes – No	-0.001	0.178	-0.007	0.994	0.99	(0.70, 1.41)
Victim of/witnessed violence						
Yes – No	0.5434	0.162	3.343	<0.001	1.72	(1.25, 2.36)
COVID-19						
Yes – No	0.161	0.090	1.773	0.076	1.17	(0.98, 1.40)
Learning disability						
Yes – No	-0.036	0.160	-0.229	0.819	0.96	(0.70, 1.32)
Developmental delay						
Yes – No	0.393	0.193	2.032	0.042	1.48	(1.01, 2.16)
Autism						
Yes – No	0.836	0.200	4.165	<0.001	2.30	(1.55, 3.42)
Intellectual disability						
Yes – No	0.325	0.290	1.121	0.262	1.38	(0.78, 2.44)
ADHD						
Yes – No	0.753	0.120	6.249	<0.001	2.12	(1.67, 2.69)
Asthma						
Yes – No	0.113	0.127	0.885	0.376	1.12	(0.87, 1.43)
Health Status						
Poor – Good	1.196	0.210	5.686	<0.001	3.30	(2.19, 4.99)

LASSO, least absolute shrinkage and selection operator; SE, standard error.

**Figure 3:**
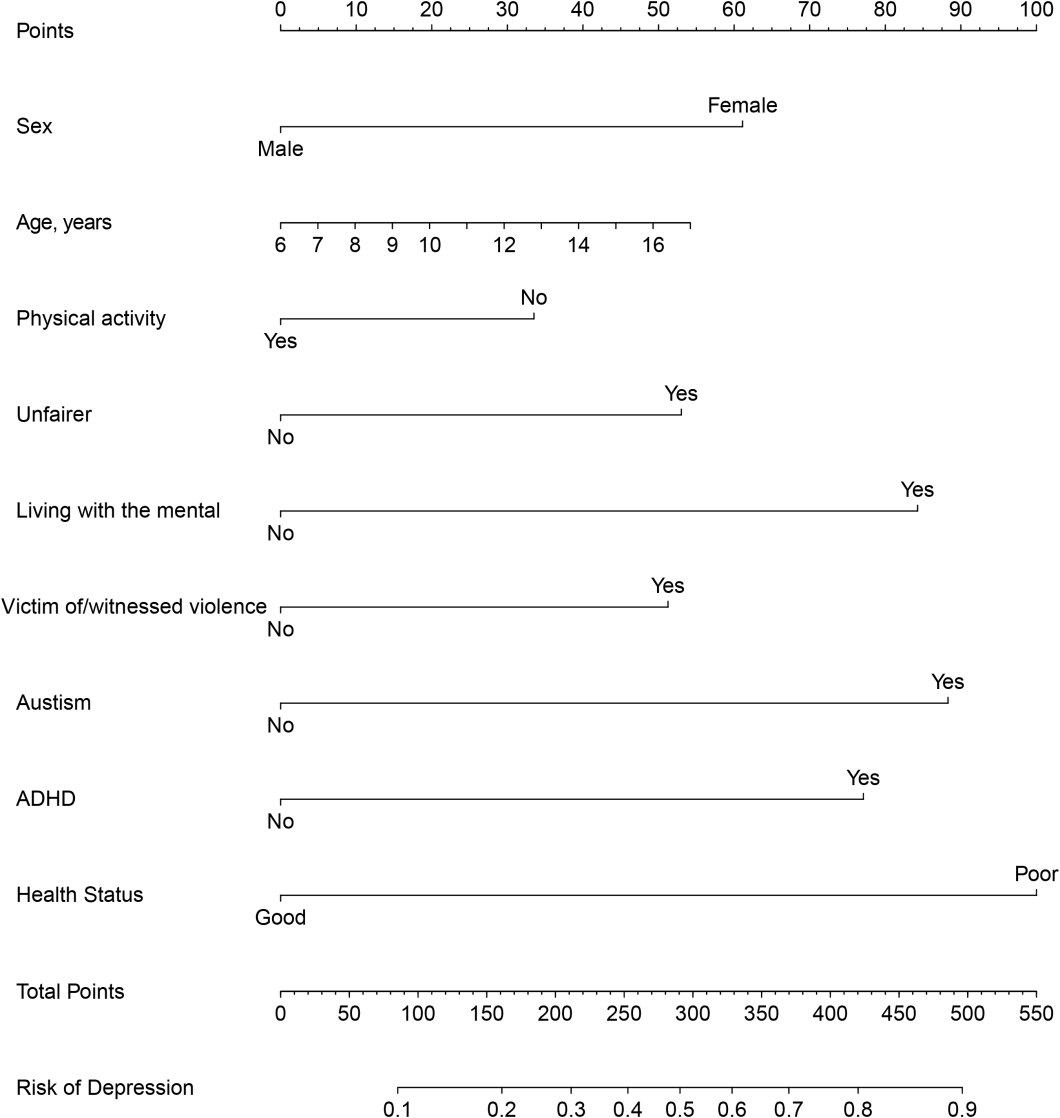
Nomogram prediction model. .

To improve the convenience of model application, we further select important indicators through multi-factor logistic screening (**Table 3**). The final logistic model included 9 independent predictors (Sex, Age, Physical activity, Unfairer, Living with the mental, Victim of/witnessed violence, Autism, ADHD, Health Status) and was developed as a simple-to-use nomogram, which is illustrated in **Figure 3**.

**Table 3:** Results of univariate and multivariate logistic regression of the final modeling variables.

Dependent: Depression		OR (univariable)	OR (multivariable)
**Sex**	Girls	-	-
	Boys	0.47 (0.40-0.57, p<0.001)	0.48 (0.40-0.57, p<0.001)
**Age, years**	Mean (SD)	1.06 (1.03-1.08, p<0.001)	1.06 (1.03-1.09, p<0.001)
**Physical activity**	Yes	-	-
	No	1.47 (1.23-1.77, p<0.001)	1.46 (1.22-1.74, p<0.001)
**Unfairer**	No	–	–
	Yes	2.13 (1.55-2.93, p<0.001)	2.14 (1.56-2.93, p<0.001)
**Living with the mental**	No	–	–
	Yes	2.03 (1.52-2.63, p<0.001)	2.08 (1.60-2.68, p<0.001)
**Victim of/witnessed violence**	No	–	–
	Yes	1.72 (1.25-2.37, p<0.001)	1.78 (1.31-2.41, p<0.001)
**Autism**	No	–	–
	Yes	2.31 (1.56-3.42, p<0.001)	2.37 (1.62-3.44, p<0.001)
**ADHD**	No	–	–
	Yes	2.12 (1.68-2.69, p<0.001)	2.17 (1.72-2.72, p<0.001)
**Health Status**	Good	–	–
	Poor	3.31 (2.19-4.99, p<0.001)	3.48 (2.32-5.19, p<0.001)

**Figure 4:**
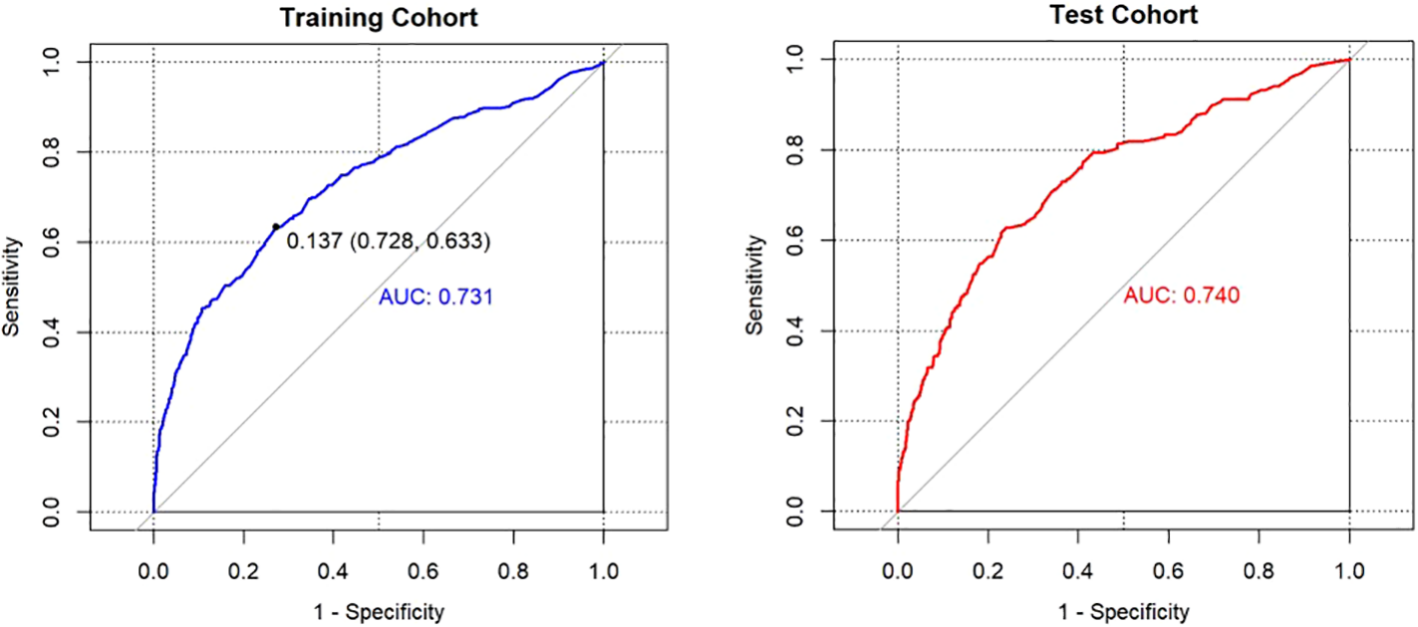
ROC curves of the nomogram prediction model in the different cohorts.

The AUCs of the model in the different cohorts were shown in the following figures (**Figure 4**). Patients in the training cohort were divided into high-risk and low-risk groups with the maximal Youden’s index as the optimal cut-off value (0.137). At this cut-off value, the prediction scores were associated with a sensitivity and specificity of 0.633 and 0.728, respectively. The AUC of the model in the internal test cohort was 0.740, indicating that the model had good generalization ability. When the optimal cut-off value determined in the training cohort was applied to the internal test cohort, the sensitivity and specificity were 0.711 and 0.657, respectively. Furthermore, the risk distribution predicted by the model in the internal test cohort showed a certain clustering of children and adolescents with or without depression (**Supplementary Figure 1**), indicating that the model accurately stratified low-and high-risk groups.

**Figure 5:**
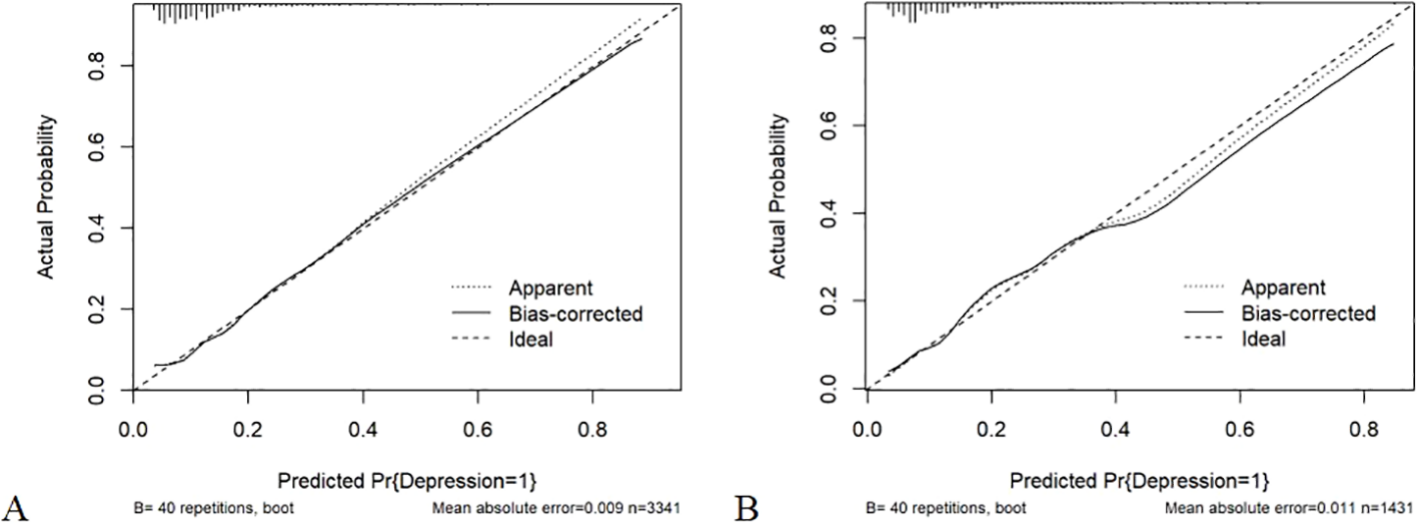
**(A)** Calibration curve of the nomogram prediction mode for the training cohort; **(B)** Calibration curve of the nomogram prediction mode for the internal test cohort.

The internal validation and calibration of the nomogram were performed using 1,000 bootstrap analyses. The calibration plots of the nomogram in the different cohorts are plotted in **Figure 5**, which demonstrate a good correlation between the observed and predicted depression. The results showed that the original nomogram was still valid for use in the test cohort, and the calibration curve of this model was relatively close to the ideal curve, which indicates that the predicted results were consistent with the actual findings.

### 3.3 Decision curve analysis

**Figure 6:**
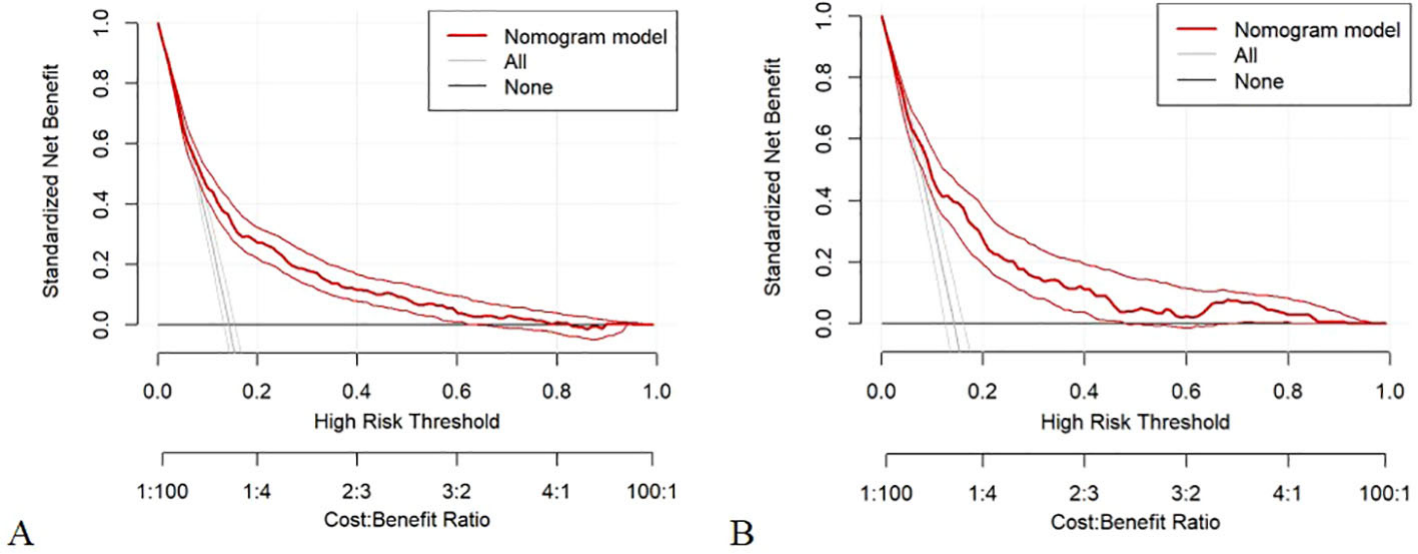
**(A)** Decision curve analysis of the nomogram of the training cohort; **(B)** Decision curve analysis of the nomogram of the internal test cohort.

**Figure 6** displays the DCA curves related to the nomogram. A high-risk threshold probability indicates the chance of significant discrepancies in the model’s prediction when clinicians encounter major flaws while utilizing the nomogram for diagnostic and decision-making purposes. This research shows that the nomogram offers substantial net benefits for clinical application through its DCA curve.

## 4 Discussion

### 4.1 Summary and comparison with existing studies

In this study, we developed and validated a diagnostic model for predicting depression in 4,772 adolescents based on factors such as clinical assessment and sociodemographic information. The main predictors included in the nomogram were gender, age, spiritual life, autism, and health status, all of which were associated with an increased risk of depression.

Overall, our findings show that girls, higher age, did not exercise, participate in sports, or engage in at least 60 minutes of physical activity daily or most days, treated/judged based on race/ethnicity, ever lived with anyone mentally ill, experienced as a victim of/witnessed violence, and ever had autism, ever had ADHD, having a poor health status may all put adolescents at increased risk for developing depression. According to previous and current studies, age is always an important variable (36). In addition, girls are a correlate of the diagnosis of depression in adolescents, which is similar to the results of previous studies (37). The steady increase in depression in girls during adolescence may be due to increased levels of estrogen and progesterone, which are sex hormones that often play an important role in emotional development (38). The current study also analyzed the impact of physical exercise on depression. Prolonged online learning in the context of an epidemic reduces the amount of time spent in physical activity (39), and those who do not exercise are more likely to have altered neuropsychiatric status than those who do, similar to the findings of previous studies (40).

However, our study uniquely highlights the importance of being treated/judged based on race/ethnicity, which has not been emphasized in other adolescent-related depression prediction studies. Treated/judged based on race/ethnicity has been recognized as a social determinant of psychological well-being (41, 42), and supporting the relationship is the Discrimination Stress, Coping, and Mental Health Framework (43). The framework argues that racial discrimination as a chronic stressor depletes an individual’s protective psychological resources, leading to a combination of risky behaviors and diminished emotional control, increasing the risk of mental health effects (43). Additionally, ever living with anyone mentally ill exacerbates family dysfunction (44) and may be an independent predictor of the development of sick mentally in adolescents (45), and even unfavorable family interactions may affect adolescents more than the presence of a parent with mental illness itself (46). Potentially traumatic experiences (PTE) include experience as a victim of/witnessed violence. Research by Annika Skandsen et al. suggests that adolescents diagnosed with depression experience approximately twice as much PTE as the reference group, which may make it possible for impaired emotion regulation due to biological changes caused by PTE to in turn trigger adolescents to overreact to stressful situations (47).

Guralnik et al. conducted sibling comparisons in Sweden and showed that compared to the general population, patients with autism spectrum disorders (ASD) were at higher risk of developing depression in young adulthood, which is similar to our results (48). ADHD is a common neurodevelopmental disorder (49), and previous studies have shown that early hyperactive-impulsive symptoms of ADHD often bring about poor emotional problems (50) and neurodevelopmental difficulties, and that one can developmentally suffer from depression (51). According to Gilles Ambresin (52), major depressive syndrome (MDS) is two times more likely to occur in people with poorer health than in those with better health, and the association remains even after adjusting for multiple diseases, gender and other factors.

### 4.2 Clinical significance of the study

Previous studies using plain Bayesian models to predict factors in Korean adolescents included only social or environmental factors and did not cover clinical factors (53). Lin Wang et al. (36) conducted a scoring study during the COVID-19 pandemic to predict psychosocial and behavioral problems in adolescents considering factors such as age, weight, and sleep problems, but the predictive benefit was low. In contrast, our proposed nomogram shows better differentiation and clinical decision-making power, visualizing the clinical factors associated with depression in adolescents and allowing better risk stratification. In addition, the nomogram helps doctors identify high-risk groups as early as possible and intervene promptly.

### 4.3 Potential limitations

Our study has several limitations that should be acknowledged. The cohort was based on patients from the US, which may not be representative of the wider population. In terms of model selection, we did not choose machine learning (ML) models. After that, our study lacks external validation from an independent cohort, which may affect the superiority and generalization ability of the model. Finally, for feature selection, we extracted some structured self-reported data but lacked imaging and genetic data.

Future research should aim to externally validate our nomogram in different populations and settings. We will try to build ML models to predict depression and integrate imaging and genetic data to enhance the predictive accuracy of the nomogram.

## 5 Conclusions

This study developed a nomogram model for predicting depression in children and adolescents with high clinical utility. We found that the sex, age, physical activity, unfairer, living with the mental, victim of/witnessed violence, autism, ADHD, health status was the main influencing factor of depression in children and adolescents.

## Data Availability

The raw data supporting the conclusions of this article will be made available by the authors, without undue reservation.
