# Corrigendum: Predictive factors for the development of depression in children and adolescents: a clinical study

**DOI:** 10.3389/fpsyt.2025.1555594

**Published:** 2025-01-24

**Authors:** Hong Zhang, Peilin Yu, Xiaoming Liu, Ke Wang

**Affiliations:** ^1^ The Second Clinical Medical School, Xuzhou Medical University, Xuzhou, Jiangsu, China; ^2^ Department of Biostatistics, School of Public Health, Xuzhou Medical University, Xuzhou, Jiangsu, China; ^3^ Xuzhou Children’s Hospital Affiliated to Xuzhou Medical University, Xuzhou, Jiangsu, China; ^4^ Center for Medical Statistics and Data Analysis, Xuzhou Medical University, Xuzhou, Jiangsu, China; ^5^ Jiangsu Engineering Research Center of Biological Data Mining and Healthcare Transformation, Xuzhou Medical University, Xuzhou, Jiangsu, China; ^6^ Research Center for Psychological Crisis Prevention and Intervention of college in Jiangsu Province, Xuzhou, Jiangsu, China

**Keywords:** logistic regression, NCHS, adolescents, depression, nomogram, prediction

In the published article, there were errors in the Affiliations. “^1^The Second Clinical Medical School, Xuzhou Medical University, Xuzhou, Jiangsu, China” was omitted for authors Hong Zhang and Xiaoming Liu. Hong Zhang was incorrectly reported as being affiliated with “Xuzhou Children’s Hospital Affiliated to Xuzhou Medical University, Xuzhou, Jiangsu, China”.

The authors apologize for these errors and state that this does not change the scientific conclusions of the article in any way. The original article has been updated.

