# Supplementary material for: Predictive factors for the development of depression in children and adolescents: a clinical study
Source: Front Psychiatry. 2024 Oct 14;15:1460801. doi: 10.3389/fpsyt.2024.1460801 (PMC11513372; doi:10.3389/fpsyt.2024.1460801)
Supplement: Supplementary file 1 [file DataSheet1.docx]

Supplementary Material

# 1 Supplementary Data

# 2 Supplementary Figures and Tables

# 2.1 Supplementary Tables

The baseline characteristics table (Supplementary table 1) provides valuable insights into the study population. In terms of age, the median (IQR) overall age was 12.0 (9.0, 15.0) years. However, when comparing those who experienced depression (Yes) to those who did not (No), there was a slightly higher median age of 14.0 (10.0, 16.0) years in the Yes group. Regarding gender, girls constituted 48% of the overall sample, whereas in the Yes group, girls accounted for a slightly higher percentage of 61%. The distribution of urban and rural dwellings, as measured by Urbanization level, did not differ significantly between the Yes and No groups (p-value = 0.124). However, a significant difference was observed in the p-values for Race/ethnicity (p < 0.001), indicating differences in the distribution of race/ethnicity across the two groups. For example, in the Yes group, 20% identified as Hispanic, whereas in the No group, the proportion was 27%. Other characteristics, such as education level (Highest level of education), disability (The Washington Group Short Set Composite Disability Indicator), substance abuse (Living with the addict), and Living with the mental, also showed significant associations with the Yes group (p < 0.001). These findings highlight the importance of considering baseline characteristics when analyzing the outcomes of interest.

Supplementary table 1. Children demographics and baseline characteristics

| **Characteristic** |  | **Depression** | | | **P-value^2^** |
| --- | --- | --- | --- | --- | --- |
|  |  | **Overall, N = 4,772^1^** | **Yes, N = 684^1^** | **No, N = 4,088^1^** |  |
| **Age, years** |  |  |  |  | <0.001 |
| Median (IQR) |  | 12.0 (9.0, 15.0) | 14.0 (10.0, 16.0) | 12.0 (9.0, 15.0) |  |
| **Sex** |  |  |  |  | <0.001 |
| Girls |  | 2,306 (48%) | 418 (61%) | 1,888 (46%) |  |
| Boys |  | 2,466 (52%) | 266 (39%) | 2,200 (54%) |  |
| **Urbanization level** |  |  |  |  | 0.124 |
| Medium and small metro |  | 1,437 (30%) | 223 (33%) | 1,214 (30%) |  |
| Large metropolitan |  | 2,676 (56%) | 359 (52%) | 2,317 (57%) |  |
| Non-metropolitan |  | 659 (14%) | 102 (15%) | 557 (14%) |  |
| **Family income to poverty ratio** |  |  |  |  | 0.019 |
| <100% |  | 512 (11%) | 96 (14%) | 416 (10%) |  |
| 100–199% |  | 968 (20%) | 125 (18%) | 843 (21%) |  |
| 200–399% |  | 1,414 (30%) | 199 (29%) | 1,215 (30%) |  |
| ≥400% |  | 1,878 (39%) | 264 (39%) | 1,614 (39%) |  |
| **Race/ethnicity** |  |  |  |  | <0.001 |
| Non-Hispanic Black |  | 485 (10%) | 56 (8.2%) | 429 (10%) |  |
| Hispanic |  | 1,239 (26%) | 138 (20%) | 1,101 (27%) |  |
| Non-Hispanic White |  | 2,396 (50%) | 405 (59%) | 1,991 (49%) |  |
| Non-Hispanic other |  | 652 (14%) | 85 (12%) | 567 (14%) |  |
| **The number of adults** |  |  |  |  | 0.048 |
| 1 adult |  | 659 (14%) | 111 (16%) | 548 (13%) |  |
| ≥2 adults |  | 4,113 (86%) | 573 (84%) | 3,540 (87%) |  |
| **The number of children** |  |  |  |  | 0.016 |
| 1 child |  | 1,908 (40%) | 302 (44%) | 1,606 (39%) |  |
| ≥2 children |  | 2,864 (60%) | 382 (56%) | 2,482 (61%) |  |
| **Highest level of education^3^** |  |  |  |  | 0.010 |
| Associate degree or some college |  | 1,301 (27%) | 219 (32%) | 1,082 (27%) |  |
| High school or less |  | 1,004 (21%) | 132 (19%) | 872 (21%) |  |
| Bachelor’s degree or higher |  | 2,461 (52%) | 332 (49%) | 2,129 (52%) |  |
| **Behavior^4^** |  |  |  |  | 0.612 |
| Yes |  | 620 (13%) | 93 (14%) | 527 (13%) |  |
| No |  | 4,152 (87%) | 591 (86%) | 3,561 (87%) |  |
| **Physical activity** |  |  |  |  | <0.001 |
| Yes |  | 3,161 (66%) | 379 (55%) | 2,782 (68%) |  |
| No |  | 1,611 (34%) | 305 (45%) | 1,306 (32%) |  |
| **Head discomfort^5^** |  |  |  |  | 0.002 |
| Yes |  | 311 (6.5%) | 63 (9.2%) | 248 (6.1%) |  |
| No |  | 4,461 (93%) | 621 (91%) | 3,840 (94%) |  |
| **Unfairer** |  |  |  |  | <0.001 |
| Yes |  | 285 (6.0%) | 81 (12%) | 204 (5.0%) |  |
| No |  | 4,487 (94%) | 603 (88%) | 3,884 (95%) |  |
| **Lacking basic needs** |  |  |  |  | <0.001 |
| Yes |  | 168 (3.5%) | 49 (7.2%) | 119 (2.9%) |  |
| No |  | 4,604 (96%) | 635 (93%) | 3,969 (97%) |  |
| **Putdown** |  |  |  |  | <0.001 |
| Yes |  | 255 (5.3%) | 91 (13%) | 164 (4.0%) |  |
| No |  | 4,517 (95%) | 593 (87%) | 3,924 (96%) |  |
| **Living with the addict^6^** |  |  |  |  | <0.001 |
| Yes |  | 466 (9.8%) | 132 (19%) | 334 (8.2%) |  |
| No |  | 4,306 (90%) | 552 (81%) | 3,754 (92%) |  |
| **Living with the mental^7^** |  |  |  |  | <0.001 |
| Yes |  | 451 (9.5%) | 156 (23%) | 295 (7.2%) |  |
| No |  | 4,321 (91%) | 528 (77%) | 3,793 (93%) |  |
| **Separate with the jailers^8^** |  |  |  |  | <0.001 |
| Yes |  | 326 (6.8%) | 85 (12%) | 241 (5.9%) |  |
| No |  | 4,446 (93%) | 599 (88%) | 3,847 (94%) |  |
| **Victim of/witnessed violence** |  |  |  |  | <0.001 |
| Yes |  | 321 (6.7%) | 106 (15%) | 215 (5.3%) |  |
| No |  | 4,451 (93%) | 578 (85%) | 3,873 (95%) |  |
| **COVID-19** |  |  |  |  | <0.001 |
| Yes |  | 1,764 (37%) | 294 (43%) | 1,470 (36%) |  |
| No |  | 3,008 (63%) | 390 (57%) | 2,618 (64%) |  |
| **Learning disability** |  |  |  |  | <0.001 |
| Yes |  | 434 (9.1%) | 108 (16%) | 326 (8.0%) |  |
| No |  | 4,338 (91%) | 576 (84%) | 3,762 (92%) |  |
| **Developmental delay** |  |  |  |  | <0.001 |
| Yes |  | 221 (4.6%) | 66 (9.6%) | 155 (3.8%) |  |
| No |  | 4,551 (95%) | 618 (90%) | 3,933 (96%) |  |
| **Autism** |  |  |  |  | <0.001 |
| Yes |  | 175 (3.7%) | 58 (8.5%) | 117 (2.9%) |  |
| No |  | 4,597 (96%) | 626 (92%) | 3,971 (97%) |  |
| **Intellectual disability** |  |  |  |  | <0.001 |
| Yes |  | 88 (1.8%) | 31 (4.5%) | 57 (1.4%) |  |
| No |  | 4,684 (98%) | 653 (95%) | 4,031 (99%) |  |
| **ADHD^9^** |  |  |  |  | <0.001 |
| Yes |  | 608 (13%) | 175 (26%) | 433 (11%) |  |
| No |  | 4,164 (87%) | 509 (74%) | 3,655 (89%) |  |
| **Asthma** |  |  |  |  | 0.003 |
| Yes |  | 609 (13%) | 111 (16%) | 498 (12%) |  |
| No |  | 4,163 (87%) | 573 (84%) | 3,590 (88%) |  |
| **Health Status** |  |  |  |  | <0.001 |
| Good |  | 4,642 (97%) | 628 (92%) | 4,014 (98%) |  |
| Poor |  | 130 (2.7%) | 56 (8.2%) | 74 (1.8%) |  |

NOTE: ^1^n (%).

^2^Wilcoxon rank sum test; Pearson's Chi-squared test.

^3^The highest level of education refers to the highest level of education among all sample child’s parents.

^4^The Washington Group Short Set Composite Disability Indicator.

^5^Ever headache, vomit, blurred vision, or mood change after blow to head.

^6^Ever lived with anyone with alcohol/drug problem.

^7^Ever lived with anyone mentally ill/severely depressed

^8^Ever separated from parent who was incarcerated.

^9^Had Attention-Deficit/Hyperactivity Disorder (ADHD) or Attention-Deficit Disorder (ADD).

Supplementary table 2. The coefficients of Lasso regression analysis

| Coefficient | variable |
| --- | --- |
| 0.51910807 | (Intercept) |
| 0.00000000 | URBRRL_level_Medium and small metro |
| 0.00000000 | URBRRL_level_Large metropolitan |
| -0.06732403 | RATCAT_C_level_100–199% |
| 0.00000000 | RATCAT_C_level_200–399% |
| 0.00000000 | RATCAT_C_level_≥400% |
| -0.38682712 | SEX_C_level_Male |
| 0.02549935 | AGEP_C_level_ |
| -0.01262861 | HISPALLP_C_level_Non-Hispanic Black |
| -0.16693517 | HISPALLP_C_level_Hispanic |
| 0.00000000 | HISPALLP_C_level_Non-Hispanic other |
| 0.00000000 | PCNT18UPTC_level_≥2 adults |
| 0.00000000 | PCNTLT18TC_level_≥2 children |
| 0.00000000 | MAXEDUCP_C_level_High school or less |
| 0.00000000 | MAXEDUCP_C_level_Bachelor’s degree or higher |
| 0.00000000 | DISAB5_C_level_No |
| 0.20951246 | PADAYS_C_level_No |
| 0.00000000 | TBIHEADSYM_C_level_No |
| -0.31210335 | UNFAIRRE_C_level_No |
| 0.00000000 | BNEEDS_C_level_No |
| -0.14146475 | PUTDOWN_C_level_No |
| 0.00000000 | ALCDRUGEV_C_level_No |
| -0.71740695 | MENTDEPEV_C_level_No |
| 0.00000000 | JAILEV1_C_level_No |
| -0.36234131 | VIOLENEV_C_level_No |
| 0.00000000 | CVDDIAG_C_level_No |
| -0.14621819 | LDEV_C_level_No |
| -0.07178288 | DDEV_C_level_No |
| -0.49500460 | ASDEV_C_level_No |
| 0.00000000 | IDEV1_C_level_No |
| -0.57681119 | ADHDEV_C_level_No |
| 0.00000000 | ASEV_C_level_No |
| 0.84557537 | PHSTAT_C_level_Poor |

# 2.2 Supplementary Figures


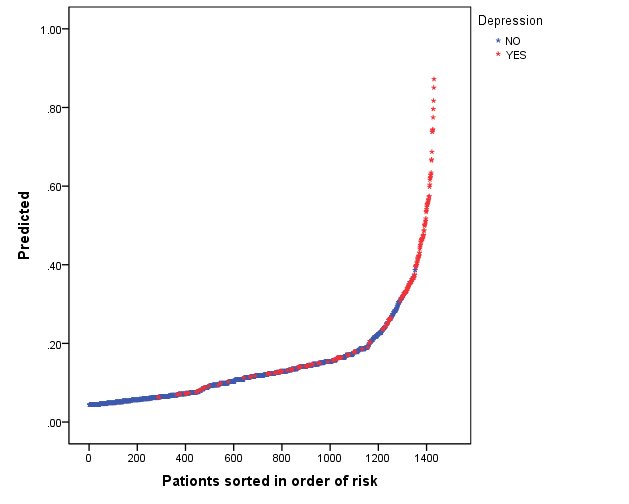


Supplementary figure 1. Prediction distributions for children and adolescents in the internal test cohort.
